# Prey Capture and Phagocytosis in the Choanoflagellate *Salpingoeca rosetta*

**DOI:** 10.1371/journal.pone.0095577

**Published:** 2014-05-07

**Authors:** Mark J. Dayel, Nicole King

**Affiliations:** 1 Department of Molecular and Cell Biology, University of California, Berkeley, Berkeley, California, United States of America; 2 Howard Hughes Medical Institute, University of California, Berkeley, Berkeley, California, United States of America; University of Hull, United States of America

## Abstract

Choanoflagellates are unicellular and colonial aquatic microeukaryotes that capture bacteria using an apical flagellum surrounded by a feeding collar composed of actin-filled microvilli. Flow produced by the apical flagellum drives prey bacteria to the feeding collar for phagocytosis. We report here on the cell biology of prey capture in rosette-shaped colonies and unicellular “thecate” or substrate attached cells from the choanoflagellate *S. rosetta*. In thecate cells and rosette colonies, phagocytosis initially involves fusion of multiple microvilli, followed by remodeling of the collar membrane to engulf the prey, and transport of engulfed bacteria into the cell. Although both thecate cells and rosette colony cells produce ∼70 nm “collar links” that connect and potentially stabilize adjacent microvilli, only thecate cells were observed to produce a lamellipod-like “collar skirt” that encircles the base of the collar. This study offers insight into the process of prey ingestion by *S. rosetta*, and provides a context within which to consider potential ecological differences between solitary cells and colonies in choanoflagellates.

## Introduction

**Figure 1:**
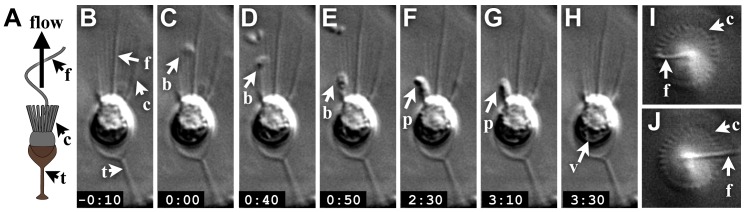
The process of prey capture and ingestion by thecate cells. (A) Schematic of a thecate cell. (B–G) A time series from a time-lapse movie of a thecate cell (Movie S1) shows phagocytosis of a bacterium at the base of the collar. A bacterial cell arrived at the collar at t = 0:00 (C), then moved around the collar and reached the base (E) where it remained for ∼2 minutes before being phagocytosed (F, G) and transported into the cell (H). (I, J) The *S. rosetta* flagellum strikes the collar as it undulates, as revealed by two images selected from a top-view time-lapse S3 of a thecate cell (imaged by DIC). Key: f  =  flagellum, c  =  collar, t  =  theca, b  =  bacterium, p  =  phagocytic cup, v  =  food vacuole.

The closest living relatives of animals, the choanoflagellates, offer an opportunity to investigate the potential connections between prey capture, multicellularity, and animal origins [1]–[4]. Choanoflagellates prey upon bacteria and, while all species have a single-celled stage to their life history, some are also capable of forming simple multicelled colonies. Whether part of a colony or unicellular, each choanoflagellate cell bears a single apical flagellum that is surrounded by a feeding collar composed of actin-filled microvilli (Fig. 1A, B; [5]–). The undulation of the apical flagellum creates fluid currents that draw bacteria into the feeding collar for phagocytosis [8], [9]. This cell morphology is conserved in the feeding cells of sponges [5], [10]–[13] and resembles that of eumetazoan epithelial cells, which are characterized by apical microvilli, a single apical primary cilium per cell, and frequently, interactions with bacteria.

Although all choanoflagellates have a unicellular phase to their life history, some species also form rosette-shaped colonies in which each cell is arranged radially around a central point, with its flagellum and collar pointing outward into the aquatic environment [14], [15]. Rosette colonies, which swim freely in the water column, offer an opportunity to investigate the connection between multicellularity and prey capture. The feeding currents created by attached solitary choanoflagellate cells, which have been measured and modeled [8], [9], [16], pull water and bacteria into contact with the outer surface of the collar. Prey bacteria subsequently become trapped against the surface of the collar, although it is not clear whether this process is solely the result of fluid flow or whether there are adhesive molecules on the surface of the collar microvilli. After capture on the collar of microvilli, bacterial prey are phagocytosed. Prior studies of the choanoflagellates *Codosiga gracilis* and *Choanoeca perplexa* have suggested that captured bacteria are encased in pseudopods [7], [17], [18], although it is uncertain whether the pseudopods originate solely from the cell body or whether collar microvilli might also contribute to the formation of phagocytic structures in choanoflagellates. In addition, it is unknown whether the mechanisms of prey capture in these two species are conserved in other choanoflagellates.

We report here on prey capture in the choanoflagellate *Salpingoeca rosetta.* The life history of the choanoflagellate *S. rosetta* includes single-celled and rosette-shaped colonial forms [14] and thus may be a good model for investigating the connections between multicellularity and prey capture. One type of solitary cell, the thecate cell, adheres to substrata by producing an organic goblet-shaped structure (the “theca”) that holds the cell several microns from environmental surfaces, orienting the cell's flagellum toward the water column. In contrast, *S. rosetta* rosette colonies are free-swimming and consist of tightly packed spheres of polarized cells in which the apical flagellum of each cell is oriented outward. We describe here the process by which captured bacterial prey are ingested, the ultrastructure of the *S. rosetta* feeding apparatus, and similarities and differences in the cell biology of prey ingestion by solitary cells and by rosette colonies.

## Results

### An overview of the dynamics and process of prey capture

Through direct observation of prey capture in *S. rosetta* thecate cells, we find that the process reproducibly involves four steps: (1) initial contact between the bacterial cell and the choanoflagellate feeding collar, (2) movement of the bacterial cell to the base of the feeding collar, (3) production of a phagocytic vesicle to surround the bacterium, and (4) phagocytosis, leading to internalization of the bacterium (Fig. 1B–H, Movie S1). After first making contact with the choanoflagellate feeding collar, the movement of the bacterial prey down the feeding collar took 12.5s on average (n = 8).

If bacteria are transported down the collar by motor-driven transport (i.e. myosin along the actin filaments in the microvilli), we would expect to see bacteria move strictly in an apical-to-basal direction along the microvilli. However, on occasion, we observed bacteria that moved laterally around the collar (i.e. traversing multiple microvilli rather than tracking along a single microvillus) as they descended toward the collar base. This suggests that motor-driven transport alone cannot explain the movement of captured bacteria to the base of the collar.

Once each captured bacterium reached the collar base, a refractile mass appeared to extend from the choanoflagellate over an average period of 20s (n = 8) to engulf the bacterium (Fig. 1F). Subsequently, the captured bacteria were transported into the cell and moved to the cell's base, where the food vacuole is located (Fig. 1G, H). Consistent with previous reports [19], [20], we also occasionally observed egestion of material from the apical surface of the cell from inside the diameter of the collar (Movie S2).

### Thecate cells: feeding structures, phagocytosis, and recruitment of bacterial prey

**Figure 2:**
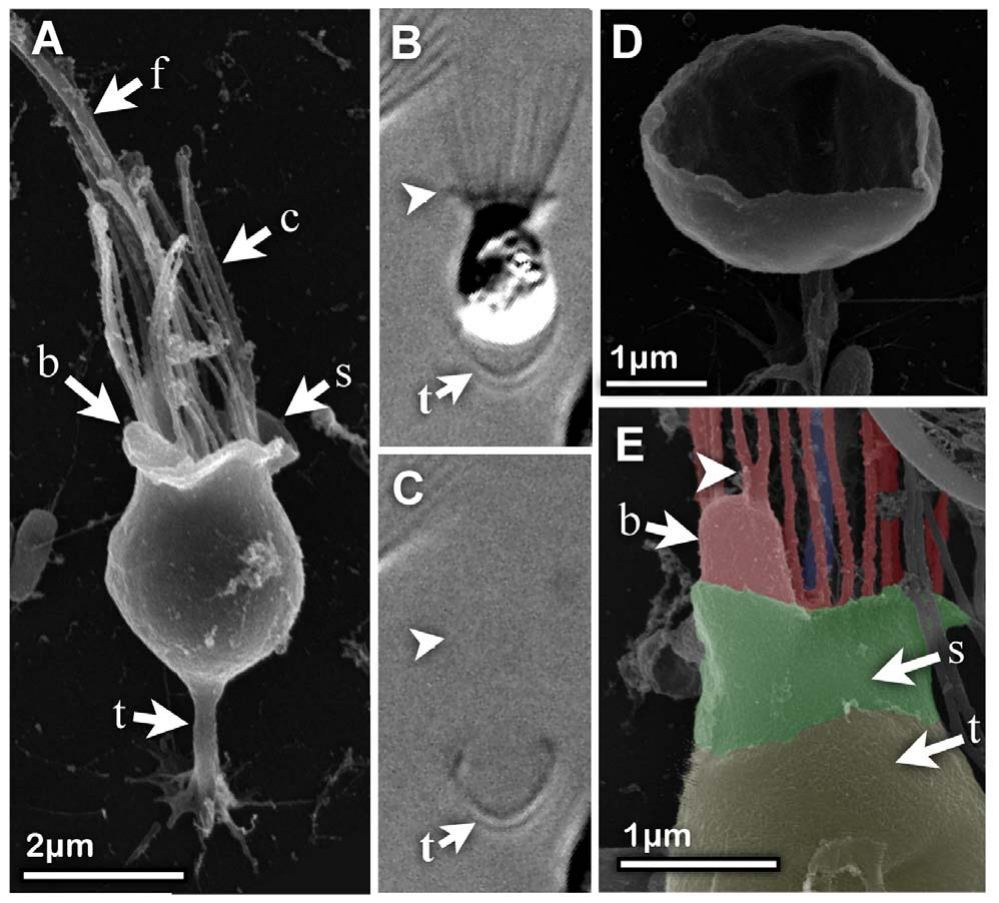
Thecate cells form a membranous collar skirt at the base of the feeding collar. (A) SEM image of a thecate cell shows a bacterium lodged between the collar and a flared “skirt” that surrounds the collar base. The collar skirt (indicated by arrowhead) was visible by light microscopy of a live cell (B). When the cell in panel (B) abandoned its theca, the flared collar skirt departed with it (C), suggesting that the collar skirt is an extension of the cortical cell membrane, rather than being an extension of the theca. (F) An SEM image of an empty theca reveals that it lacks a collar skirt. (G) An SEM image shows a bacterial cell after phagocytosis but before being drawn into the cell. The bacterium is nested inside the collar microvilli and the two microvilli above it have fused (indicated by arrowhead). SEM image is false colored red to emphasize the continuity of the microvillar membrane with the membrane covering the bacterial cell. The color skirt is colored bright green and the theca is colored olive green. Key: f  =  flagellum, c  =  collar, t  =  theca, b  =  bacterium, s  =  skirt.

To investigate the cell biological bases of prey capture and ingestion in *S. rosetta*, we used a combination of live cell imaging, TEM, and SEM in thecate cells and rosette colonies. Thecate cells attach to the substratum via a theca (a goblet-shaped structure composed of secreted organic material) that stabilizes the cell body at a distance of ∼3 microns from the substratum and orients the cell orthogonal to the surface so its flagellum points into the water column [14]. Using time-lapse video microscopy, we found that the apical flagellum in thecate cells strikes the collar as it beats from side to side (Fig. 1I and J). The flagellum has previously been observed to beat sinusoidally in a plane [14] thereby generating fluid currents that draw bacteria into contact with the collar. Through the use of TEM, we observed captured bacteria lodged between the collar and a lamellipod-like “collar skirt” that surrounds the outer base of the collar (Fig. 2 and S1). This collar skirt was observed to be either pressed flat against the collar or angled up to 45° away (Fig. 2A, E).

To determine whether the collar skirt is an extension of the theca (i.e. composed of extracellular matrix) or of the cortical cell membrane, we examined the collar skirt in live cells. Using DIC light microscopy, we found that the collar skirt in some cells is visible as a short structure at the collar base (Fig. 2B). When the thecate cell abandons its theca, the collar skirt remains with the cell, and not with the theca (Fig. 2C,D), suggesting that the collar skirt is an extension of the cell body and not continuous with the theca.

Although captured bacteria can be seen lodged between the collar skirt and the base of the microvillar collar, we found no evidence that the skirt directly engulfs bacteria. Instead, in cells that have captured bacteria, the microvilli were frequently observed to be fused above the phagocytosed bacterium (Fig. 2E) indicating that microvillar collar itself, and not the skirt, phagocytoses the bacteria.

**Figure 3:**
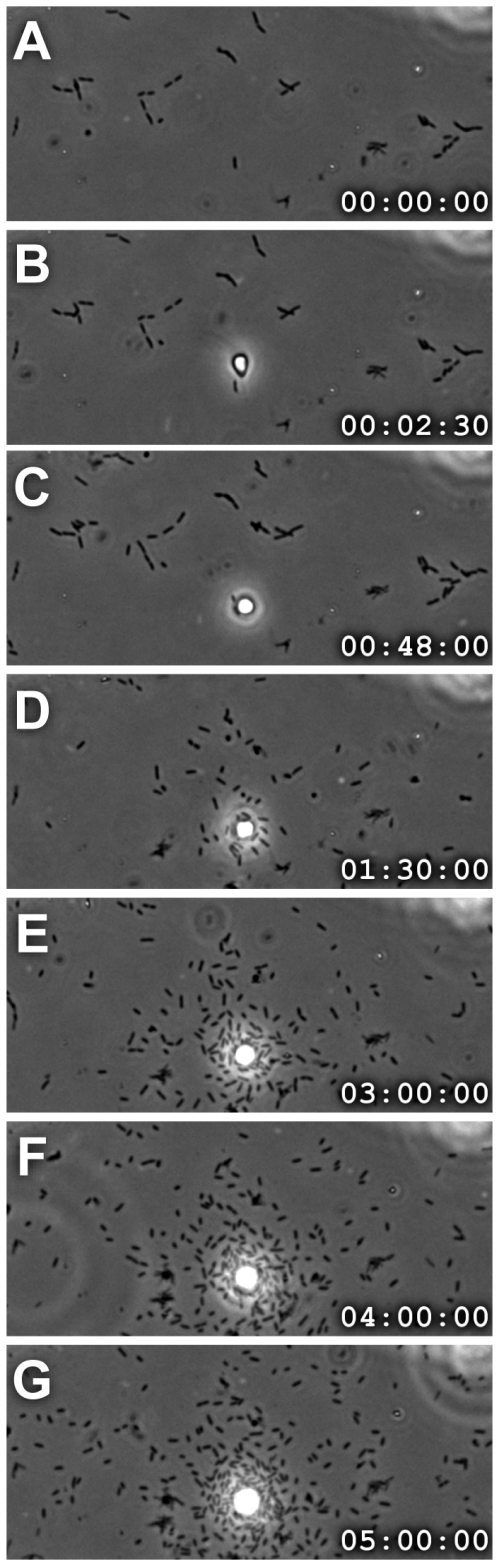
Thecate cells recruit bacteria to environmental surfaces surrounding the base of the theca stalk. A time series taken from Movie S3 of a thecate cell reveals how the presence of a choanoflagellate cell can influence the distribution of bacteria. On an environmental surface unoccupied by choanoflagellates, the bacteria were distributed randomly (see phase dark rods in panel A). (A–C) Afterward, a *S. rosetta* fast swimmer cell arrived at the previously unoccupied surface (B, phase bright cell at center of frame), attached, and differentiated into a thecate cell (C–D). After differentiation, the flagellum began to beat and bacteria were drawn toward the choanoflagellate, resulting in cluster of surface bacteria around the base of the theca (D–G). (time stamps show hours:minutes:seconds).

The flow produced by thecate cells had the unexpected effect of gathering bacteria onto the environmental surfaces around the thecate cell. Fig. 3 and Movie S3 illustrate how the arrival of a choanoflagellate altered the density and distribution of surface bacteria. Before the choanoflagellate arrived, surface bacteria were observed as dark specks sparsely and randomly distributed across the surface (Fig. 3A). Once the choanoflagellate attached to the surface (Fig. 3B), no significant bacterial transport was observed for the next ∼45 minutes while the cell differentiated from a fast swimmer into a thecate cell (Fig. 3C) [14]. Over the subsequent ∼45 minutes, however, bacteria were drawn towards the choanoflagellate, and many were deposited on the surface close to the cell (Fig. 3D). Over the next several hours, this flow-driven transport (combined, presumably, with bacterial cell division) increased the density of surface-attached bacteria around the choanoflagellate (Fig. 3E–G).

### Rosette colonies: feeding structures and phagocytosis

Like thecate cells, cells in rosette colonies use the apical flagellum to generate currents that draw bacteria to the feeding collar. However, in contrast with thecate cells, we find that cells in rosette colonies lack a collar skirt at the base of the collar. This indicates a potentially important biological difference between thecate cells and rosette colonies. The lack of a skirt also offers a less obstructed view of the process of phagocytosis.

**Figure 4:**
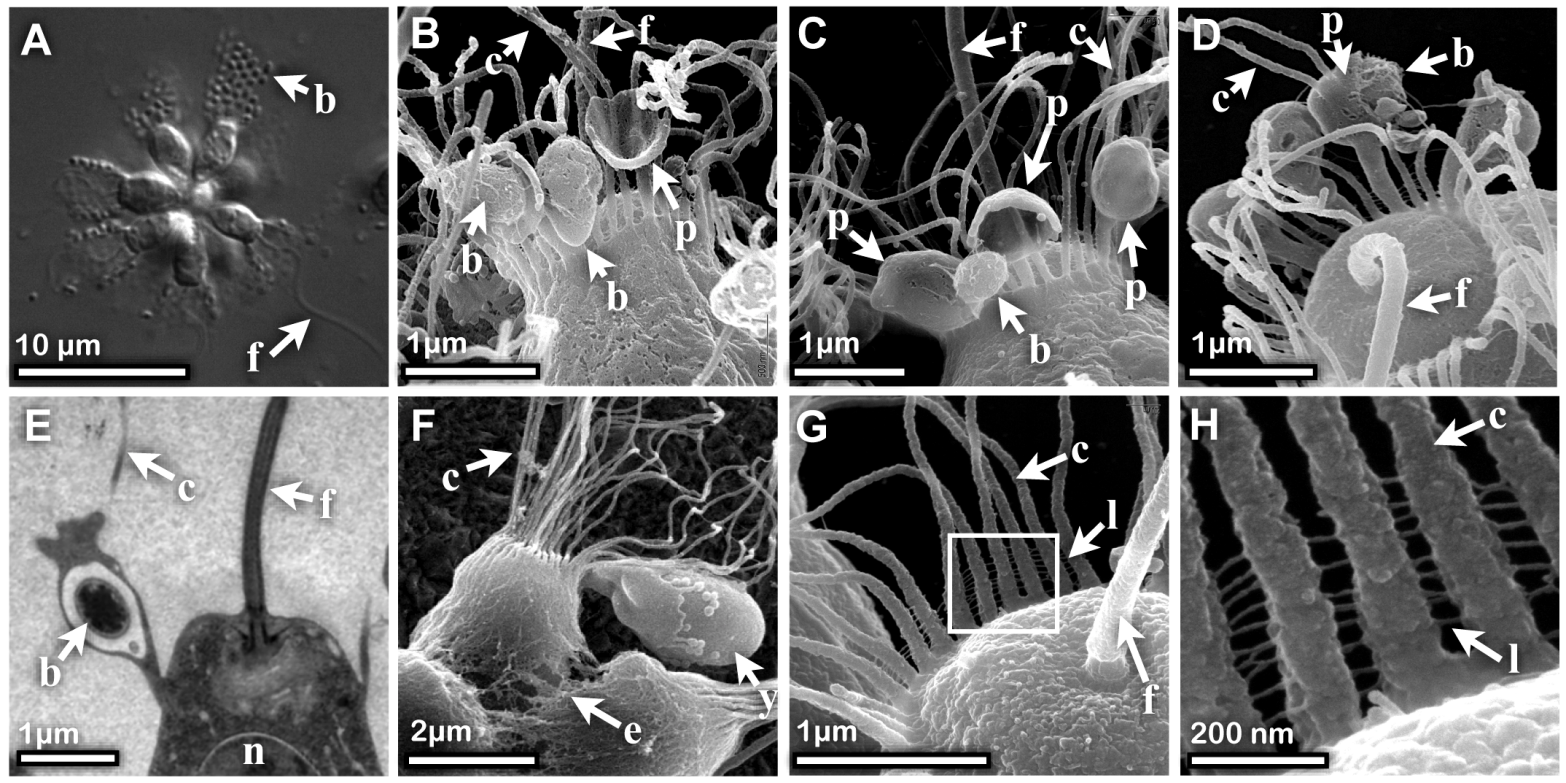
Phagocytosis of bacteria and yeast by rosette colonies. (A) DIC image of live *S. rosetta* colony showing that under conditions of high bacterial cell density, the bacteria pack tightly along the lengths of the collars of rosette colony cells. (B, C) SEM images of two cells from rosette colonies show that phagocytic cups form directly from the collar ∼700 nm from the collar base. (D) Bacteria are engulfed directly into collar microvilli that are often fused together, as revealed by SEM imaging of a rosette colony cell. (E) A TEM image of phagocytosis shows a bacterium that has been engulfed by membrane associated with the feeding collar membrane. (F) SEM image shows phagocytosis of *Saccharomyces cerevisiae*. (G, H) SEM images of the links between adjacent microvilli at the base of the collar, with (H) showing higher magnification of square in (G). Key: f  =  flagellum, c  =  collar microvilli, b  =  bacterium, p  =  phagocytic cup, y  =  *S. cerevisiae*, e  =  extracellular matrix, l  =  collar links.

When rosette colonies were grown in the presence of high concentrations of bacteria, densely packed bacteria were observed to fully cover the collars (Fig. 4A). In *S. rosetta* rosette colony cells, phagocytic cups appeared to form directly from the microvilli and grow to surround prey bacteria (Fig. 4B). Once engulfed, the prey bacteria became encased within a club-like structure on the collar (Fig. 4C–E). At its base, the phagocytic structure was thicker than a single microvillus and often displayed two microvilli protruding from the swelling containing the bacterium (Fig. 4D), suggesting that it formed from the fusion of multiple microvilli. This mechanism of phagocytosis is capable of capturing remarkably large prey, including the yeast *Saccharomyces cerevisiae* (Fig. 4F).

We typically found that phagocytosis of bacteria was confined to a region about ∼700 nm from the collar base (Fig. 4B–D, S2A and B). Within the basal-most 500 nm of the collar, we detected ∼70 nm long “links” connecting adjacent microvilli (Fig. 4G, H). We also observed similar links at the base of the collar in *Monosiga brevicollis* (Fig. S2C) where the cadherin MBCDH1 is localized [21]. These findings, along with the resemblance of these links to cadherin-based tip links in hair cells [22], raised the possibility that the links might be cadherins. However, we found that they were not eliminated by chelation of free calcium with EGTA (see Methods), suggesting that the links are either composed of cadherins that are insensitive to calcium chelation or are not cadherins (Fig. S2D). Thus, the composition of these links and their possible relation to the phagocytic functions of the collar remain unclear.

## Discussion

### Bacterial transport by fluid flow

We report a number of factors that differentiate prey capture and phagocytosis in thecate cells and rosette colonies. Thecate cells produce a collar skirt whose function and impact on prey capture are unknown. In addition, the fluid flows generated by thecate cells led to the accumulation of bacteria near the base of the theca. In contrast, colonial cells lack a skirt and capture prey while tumbling through the water column.

One surprising observation from our study relates to the fact that the transport of bacteria to the base of the collar does not seem to rely on molecular motors. Instead, we hypothesize that flowing water may contribute to bacterial transport. This inference may seem surprising given that the large-scale flow-field (over tens of microns) shows water flowing away from the collar base [8], [9]. However, if water flow through the collar dominates at regions very close (∼microns) to the collar, this may contribute to the movement of bacteria toward the collar base once they are in contact with the collar. Although beyond the scope of this study, future comparisons of the trajectories of different sized particles (i.e. those that can pass through the collar microvilli vs. those that can't) may help to illuminate the role of local flow fields in the transport of captured bacteria toward the base of the collar.

An important factor when considering the efficiency of prey capture (ratio of the number of prey captured to the number encountered, e.g. [23]) is the retention of bacteria that have contacted the collar of microvilli on a choanoflagellate. In both *S. rosetta* single cells and colonies, bacteria are often drawn into contact with the collar, remain there for a few seconds to tens of seconds, and then are swept away, slipping off the end of the collar without being phagocytosed. In contrast, loricate choanoflagellates such as *Diaphanoeca grandis* funnel the inflow of water through a veil attached to their lorica [24], and thus may exhibit a higher capture efficiency than *S. rosetta.*

### Cell Biology of Phagocytosis

Our data suggest that phagocytosis in *S. rosetta* occurs directly on the collar. What remains to be determined is whether this mode of prey capture is universal in choanoflagellates. Images from previous studies of prey capture by *Codosiga gracilis* and *Choanoeca perplexa* suggested that a pseudopodium extends from the protoplast by advancing alongside the collar tentacles [17], [18]. Notably, Plate 4E of reference [18] taken from *Codosiga gracilis* shows what appears to be a pseudopodium with two fused microvilli extending from its top. These data are consistent with our observations (Fig. 4D), suggesting that the pseudopodium and the collar are not separate structures and that phagocytosis may be occurring directly on the collar in *C. gracilis* and *C. perplexa*. Indeed, it is possible that *C. gracilis, C. perplexa* and *S. rosetta*, and potentially all choanoflagellates, share a common mechanism for engulfing bacterial prey by phagocytosis on the feeding collar.

### Collar links

We report here that thin, regularly spaced collar links connect neighboring microvilli in *S. rosetta* and *M. brevicollis*. While the functions of the links remain unclear, they could contribute to the regular spacing and alignment of collar microvilli that are observed in live cells. Fjerdingstad [13] previously reported observing ‘irregular strands’ of material between collar microvilli of *Codonosiga botrytis* in TEM micrographs. These structures may be related to the collar links we observe by SEM in *S. rosetta* and *M. brevicollis*, suggesting that collar links may be a general feature of the choanoflagellate collar. This similarity may also extend to sponge choanocytes, which also possess collar links. In *E. fluviatilis*, these links, described as a glycocalyx, extend the length of the collar [25], [26]. Indeed, it is possible that *S. rosetta* collar links normally extend the entire length of the microvilli in live cells, but are disrupted by shear forces along the distal regions of the microvilli during processing for SEM. Collar links (or the glycocalyx) in sponge choanocytes, in contrast, may be protected from shear forces because the choanocytes are embedded in other sponge cell layers.

### Collar skirt

Our data reveal that the base of the feeding collar in *S. rosetta* is surrounded by a lamellipod-like extension of the cell membrane called the collar skirt. While the function of the collar skirt is not known, it may be a general feature of choanoflagellates. The choanoflagellate genus *Diplosiga* has been characterized by the presence of a short, second collar at the base of the main collar [19], [27]. These descriptions of *Diplosiga* are based on light micrographs, and we suggest that this second collar may in fact be a skirt similar to that we observe in *S. rosetta* by light and electron microscopy. Furthermore, the positioning and morphology of the *S. rosetta* collar skirt resemble the rim of the thecae in choanoflagellate species such as *Salpingoeca urceolata* that have flask-shaped thecae whose rims extend over the base of the collar [1]. It is possible that the flared flask shape conferred by the collar skirt in combination with the simple theca in *S. rosetta* may serve a similar hydrodynamic function to the flask-shaped thecae of other species. It is notable that cells in *S. rosetta* rosette colonies lack the collar skirt and this may impact both the hydrodynamics and prey capture of colonies.

### Future questions

This study describes the general feeding strategies of *S. rosetta*, including transport of the bacteria to the base of the collar and phagocytosis on the collar. In addition, we report differences in the cell biology of thecate cells and rosette colonies that may influence the ability of these two life stages of choanoflagellates to capture bacterial prey. In the future it will be important to determine (1) the function of the collar skirt, (2) the function and composition of the collar links, and (3) how cell morphology (e.g. the presence or absence of the collar skirt) affects the feeding current and prey capture. Furthermore, phagocytosis in choanoflagellates is triggered by many substances (e.g. diverse bacteria, latex beads, and yeast), suggesting a lack of selectivity in prey capture. Therefore, a challenge for the future is to determine how phagocytosis is initiated and how or if choanoflagellates differentiate between prey, pathogen, and conspecifics. Ultimately, it will be interesting to determine the ecological implications of rosette colony formation vs. surface attachment (i.e. theca formation) in choanoflagellates.

## Methods

### Growth media

Artificial seawater (ASW) was made by dissolving 32.9 g Tropic Marin Sea Salt (Tropic Marin, Montague, MA) into 1L distilled water and filtering the solution through a 0.2 µm filter. Growth media was produced by adding 2.5 g/L Cereal Grass Media (#9448604 Scholar Chemistry, Avon, NY) to freshly autoclaved (i.e. hot) ASW, incubating for 4 hours, and filtering the solution through #1 Whatman paper followed by a 0.2 µm filter [28].

Cells were cultured as previously described [21], [28]. To maximize the chance of observing phagocytosis, cells were processed during log-phase growth when prey bacterial concentrations were high.

### Light Microscopy

Live cells were viewed on a Leica DMI6000B Microscope using DIC or phase optics as previously described[14]. Images were recorded with a DFC350 FX camera.

### Electron Microscopy

Cells were immobilized by growing directly onto silica wafers (for *M brevicollis* and *S. rosetta* thecate cells), or spun down and fixed to silanized silica wafers for colonies (as previously described [14]). Cells were fixed either by high pressure freezing followed by freeze substitution into acetone with 0.2% uranyl acetate and 0.01% osmium (Fig. 2A,D,E and 4E,F); or by mixing 1:1 with 5% glutaraldehyde + 100 mM HEPES pH 8.0 in ASW for 20 minutes (Fig. 4B–D,G,H) followed by subsequent processing with uranyl acetate and osmium as above. Samples were then processed for SEM or TEM as previously described [14].

### EGTA treatment of collar links

Live *S. rosetta* cells were mixed 1:1 either with EGTA solution (60 mM EGTA + 100 mM NaCl_2_ in ASW) or with a control solution (60 mM EGTA + 100 mM CaCl_2_ in ASW) for 5 minutes then fixed by mixing 1∶1 with glutaraldehyde fixative as described above, before being processed double-blind for SEM imaging. Similar experiments were performed with 100 mM EGTA in ASW as the chelation condition and ASW as control for 1 minute. The different conditions did not produce any detectable differences in the prevalence of collar links.

## Supporting Information

Figure S1 Additional examples of the lamellipodial skirt. (A) Thecate cell showing skirt. (B,C) Higher resolution views showing skirt (arrowheads) to be thicker than microvilli diameter.
(TIF)

Figure S2 Additional examples of collar links (arrows) observed in *S. rosetta* (A, B) and *M. brevicollis* (C). (D) Treatment of *S. rosetta* with EGTA leads to a loss of microvillar rigidity, but does not disrupt the collar links.
(TIF)

Movie S1 DIC timelapse movie of *S. rosetta* thecate cell showing capture and phagocytosis of bacteria. Times indicated are hh:mm:ss.
(MOV)

Movie S2 DIC Timelapse movie of *S. rosetta* thecate cell showing egestion of material, transported from the food vacuole to the inside base of the collar, exiting the cell between the collar and flagellum, and carried away by the current. Times indicated are hh:mm:ss.
(MOV)

Movie S3 Phase microscopy timelapse movie showing the arrival of an *S. rosetta* thecate cell and subsequent accumulation of bacteria on coverslip surface in the region surrounding the cell. Times indicated are hh:mm:ss.
(MOV)
