# Supplementary figures and images for: Prey Capture and Phagocytosis in the Choanoflagellate Salpingoeca rosetta
Source: PLoS One. 2014 May 7;9(5):e95577. doi: 10.1371/journal.pone.0095577 (PMC4012994; doi:10.1371/journal.pone.0095577)

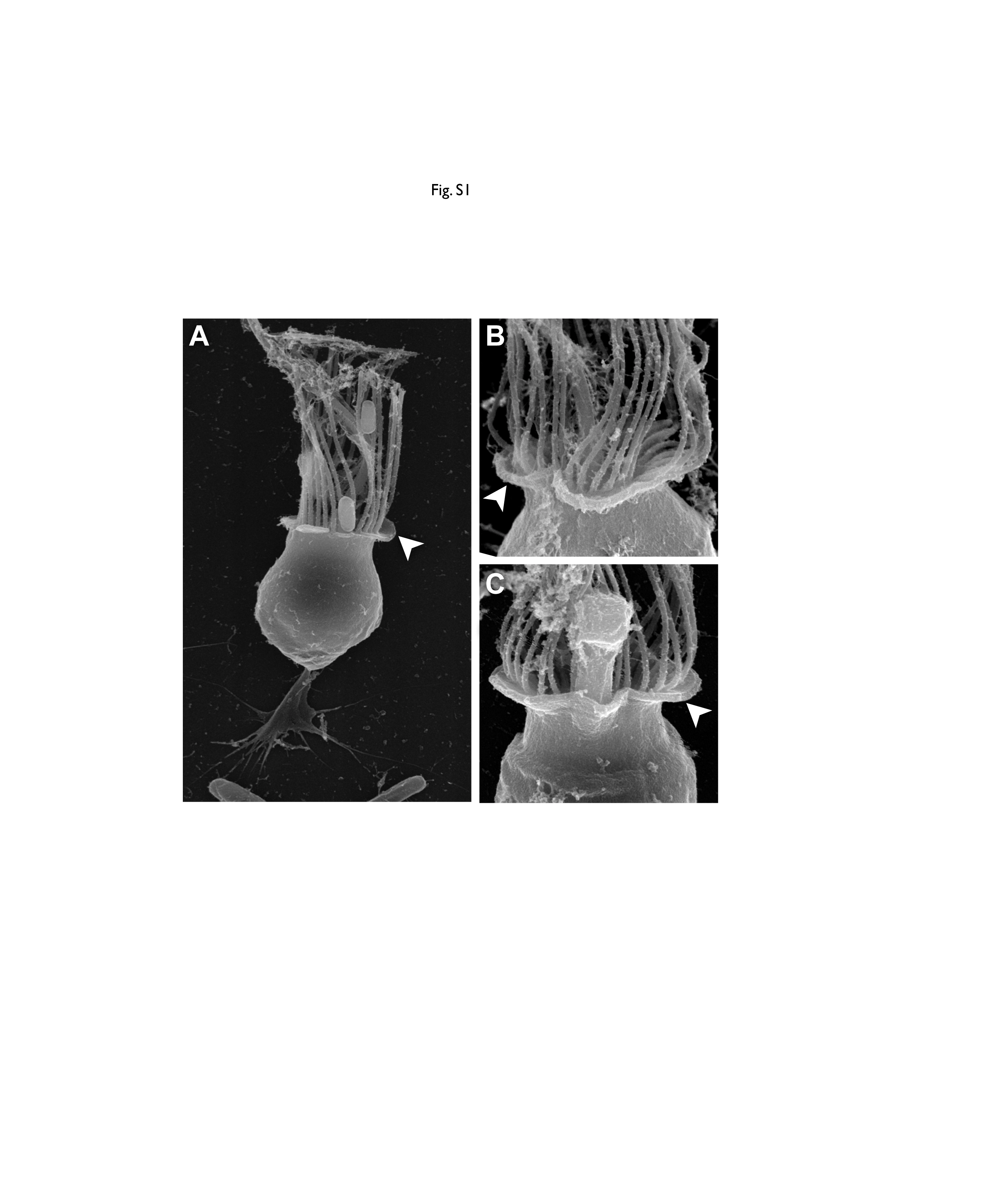

Supplement: Figure S1 — Additional examples of the lamellipodial skirt. (A) Thecate cell showing skirt. (B,C) Higher resolution views showing skirt (arrowheads) to be thicker than microvilli diameter. (TIF) [file pone.0095577.s001.tif]

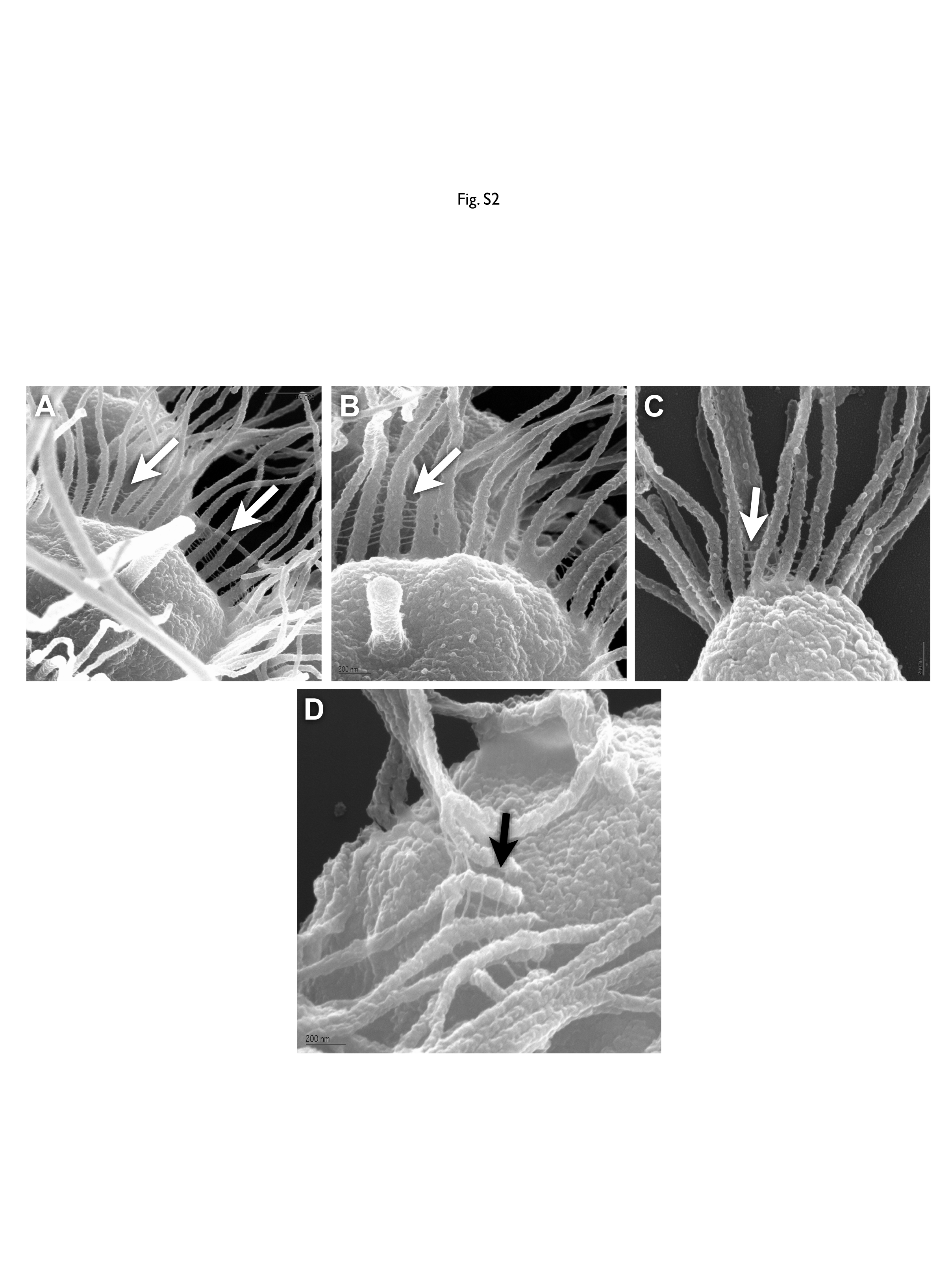

Supplement: Figure S2 — Additional examples of collar links (arrows) observed in S. rosetta (A, B) and M. brevicollis (C). (D) Treatment of S. rosetta with EGTA leads to a loss of microvillar rigidity, but does not disrupt the collar links. (TIF) [file pone.0095577.s002.tif]
